# Effect of fortification on the osmolality of human milk

**DOI:** 10.1186/s13052-023-01463-2

**Published:** 2023-06-14

**Authors:** Serena Elia, Martina Ciarcià, Nicoletta Cini, Francesca Luceri, Mauro Leucio Mattei, Francesca Miselli, Silvia Perugi, Alessandra Fanelli, Carlo Dani

**Affiliations:** 1 Division of Neonatology, Careggi University Hospital of Florence, Largo Brambilla, Florence, Italy; 2 General Laboratory, Careggi University Hospital of Florence, Florence, Italy; 3 Department of Neurosciences, Psychology, Drug Research and Child Health, University of Florence, Florence, Italy

**Keywords:** Human milk, Fortification, Osmolality, Medium-chain triglycerides

## Abstract

**Background:**

It is known that human milk fortifiers (HMF) increases osmolality of human milk (HM) but some aspects of fortification have not been deeply investigated. Our aim was to evaluate the effect of fortification on the osmolality of donor human milk (DHM) and mother’s own milk (MOM) over 72 h of storage using two commercial fortifiers and medium-chain triglycerides (MCT) supplementation.

**Methods:**

Pasteurized DHM and unpasteurized preterm MOM were fortified with 4% PreNAN FM85, 4% PreNAN FM85 plus 2% MCT, or 4% Aptamil BMF. Osmolality was measured in unfortified DHM and MOM and, moreover, just after fortification (T_0_), and after 6 (T_6_), 24 (T_24_) and 72 h (T_72_) to determine the effect of mixing and storage.

**Results:**

Unfortified DHM and MOM did not show changes of osmolality. Fortification increased osmolality of DHM and MOM without changes during the study period, except for Aptamil BMF which increased osmolality of MOM. The addition of MCT to fortified human milk (FHM) did not affect its osmolality.

**Conclusions:**

Changes of osmolality in the 72 h following fortification of both DHM and MOM did not exceed the safety values supporting the theoretically possibility of preparing 72 h volumes of FHM. Supplementation with MCT of FHM does not change osmolality suggesting that increasing energy intake in preterm infants via this approach is safe.

## Background

Human milk (HM) feeding is well recognized as the best source of nourishment both for preterm and term newborns [1]. When mother’s own milk (MOM) is insufficient, preterm infants should be supplemented with pasteurized donor human milk (DHM) [2]. In fact, it has been demonstrated that HM feeding improves cognitive outcomes, reduces risk of necrotising enterocolitis (NEC), late onset sepsis (LOS) and severe retinopathy of prematurity (ROP) in very low birth weight infants (VLBW) [3].

On the other hand, preterm HM and DHM are unable to provide the nutrient intakes recommended for fully enterally fed preterm infants [4], especially for proteins whose concentration in HM progressively declines after birth and becomes much less compared to the required 3.5–4.5 g/kg per day for VLBW.

Therefore, commercial human milk fortifiers (HMF) have been commonly used over recent years in neonatal intensive care unit (NICU) to supplement DHM and preterm HM in order to optimize the growth and outcome in very preterm infants [5]. However, while HMF increases protein and minerals content of HM, it also increases its osmolality from an isotonic ~ 300 mOsm kg^−1^ solution to a potentially hypertonic solution [6] exceeding the American Academy of Pediatrics (AAP) recommended safety cut-off value of 450 mOsm/kg [7].

Many factors can influence milk osmolality such as gestational age and postconceptional age of HM [8], diurnal cycles, storage, and heat treatment [9], but from a clinical point of view fortification remains the most important factor because an excessive osmolality has been associated to feeding intolerance and increased risk of necrotizing enterocolitis (NEC) [10]. These concerns have been dissipated by more recent studies that have excluded this correlation [5]. However, some aspects of the fortification procedure have not been deeply investigated: although it is recommended a maximum of 24 h between preparation and administration of HMF [11], it could be advantageous to prolong the storage time for FHM to 72 h, but changes of osmolality after this period of time and possible bacterial contamination have been poorly studied; moreover, the effect of the addition of lipids on osmolality of FHM has never been evaluated, although they are commonly added due to their high energy intake.

Thus, the aim of this study was to evaluate the effect of fortification on the osmolality of DHM and MOM during 72 h of storage using two commercial HMF and MCT supplementation.

## Methods

This prospective study was performed at the General Laboratory and the third level NICU of Careggi University Hospital of Florence, Italy, from June to September 2021. Formal ethical approval was not required because all analyzed milk samples were collected from mothers who had milk production 20% greater than their child’s needs and consented to donate their milk for this research.

Pasteurized DHM was donated from 15 to 365 days after term delivery, while MOM was donated from 8 to 28 days after preterm delivery which occurred < 32 weeks of gestation.

Five samples of pasteurized DHM, from pooled donors, and unpasteurized MOM, from single donors, were collected. Osmolality was measured without fortification or after fortification with 4% PreNAN FM85 (Nestlé, Vevey, Switzerland: 35.5 g protein, 32.4 g carbohydrate, and 18.1 g lipid per 100 g), 4% PreNAN FM85 plus 2% MCT Oil (95 g lipid/100 g), or 4% Aptamil BMF (Milupa, Friedrichsdorf, Germany: 25 g protein, 62 g of carbohydrate per 100 g). These supplementations are frequently used in NICU.

All donors used an electric breast pump to collect milk. Pasteurized DHM was frozen at -20 °C and stored at 4 °C for 24 h before fortification, while unpasteurized MOM was only stored at 4 °C for 24 h before fortification. Samples were heated to 37 °C for 5 min before fortification and, after the addition of fortifiers, mixtures were vortexed 1 min to homogenize them. Aliquots were stored at 4 °C and heated to 37 °C 5 min before osmolality measurements.

Osmolality, defined as the concentration of osmoles of solute per kilogram of solvent (mOsm/kg), was determined using the Advanced® Model 3320 Micro-Osmometer (Advanced Instruments Inc., MA, USA) based on freezing point. Measurements were performed just after fortification (T_0_), and after 6 (T_6_), 24 (T_24_) and 72 h (T_72_) to determine the effect of mixing and storage.

### Statistical analysis

All values are expressed as mean ± SD, the data being normally distributed. Serial measurements of mean osmolality were compared over time within the group by repeated-measures ANOVA test. Comparison of osmolality at each study point between unfortified and fortified HM was made using the Student t test. A p value of < 0.05 was considered statistically significant.

## Results

Unfortified DHM did not show changes of osmolality at T_6_, T_24_, and T_72_ in comparison with T_0_. Fortification of DHM with 4% FM85, 4% FM85 plus 2% MCT Oil, and 4% Aptamil BMF was followed by an increase of osmolality in comparison with unfortified DHM at T_0_, T_6_, T_24_, and T_72_. However, osmolality did not change over time after addition of each fortifier to DHM and there were not differences between different modes of fortification. (Table 1)

**Table 1:** Changes of osmolality (mOsm/kg) at T_0_, T_6_, T_24_, and T_72_ in unfortified pasteurized donor human milk (DHM) and in DHM fortified with 4% PreNAN FM85, 4% PreNAN FM85 and 2% MCT, and 4% Aptamil BMF. Mean ± SD.

	T_0_	T_6_	T_24_	T_72_	P
Unfortified DHM	298.6 ± 8.4	295 ± 4.1	297.2 ± 5.2	299.2 ± 5.2	0.693
DHM plus 4% FM85	422.2 ± 17.1*	426.6 ± 20.0*	431.0 ± 20.5*	434.2 ± 22.8*	0.800
DHM plus 4% FM85 and 2% MCT	422.8 ± 18.0*	430.2 ± 18.6*	433.0 ± 22.9*	438.4 ± 19.3*	0.663
DHM plus 4% Aptamil BMF	426.8 ± 16.0*	441.4 ± 15.6*	442.6 ± 18.5*	446.8 ± 18.2*	0.313

*P < 0.001 vs. No-fortified DHM.

Unfortified MOM did not show changes of osmolality at T_6_, T_24_, and T_72_ in comparison with T_0_. Fortification of MOM with 4% FM85, 4% FM85 plus 2% MCT Oil, and 4% Aptamil BMF was followed by an increase of osmolality compared to unfortified MOM at T_0_, T_6_, T_24_, and T_72_. The osmolality did not change over time in MOM fortified with FM85 and 4% FM85 plus 2% MCT and there were not differences of osmolality between these two modes of fortification. Conversely, osmolality of MOM fortified with 4% Aptamil BMF significantly increased from T_0_ to T_6_, T_24_, and T_72_, and was higher than that of MOM fortified with 4% FM85 and 4% FM85 plus 2% MCT at each study point. (Table 2)

**Table 2:** Changes of osmolality (mOsm/kg) at T_0_, T_6_, T_24_, and T_72_ in unfortified unpasteurized preterm mother’s own milk (MOM) and in MOM fortified with 4% PreNAN FM85, 4% PreNAN FM85 and 2% MCT, and 4% Aptamil BMF. Mean ± SD.

	T_0_	T_6_	T_24_	T_72_	P
Unfortified MOM	297.2 ± 4.8	301.0 ± 1.4	301.6 ± 2.1	300.6 ± 1.1*	0.092
MOM plus 4% FM85	396.2 ± 12.7*	395.0 ± 11.3*	396.2 ± 13.9*	401.4 ± 14.2*	0.848
MOM plus 4% FM85 and 2% MCT	389.0 ± 17.3*	396.0 ± 18.3*	398.0 ± 25.0*	402.0 ± 24.0*	0.809
MOM plus 4% Aptamil BMF	435.0 ± 6.5*^#^	451.2 ± 4.6*^#^	447.8 ± 9.2*^#^	459.6 ± 8.9*^#^	< 0.001

*P < 0.001 vs. No-fortified DHM.

^#^P < 0.001 vs. MOM plus 4% FM85 and 4% FM85 and 2% MCT.

The osmolality of DHM and MOM unfortified and fortified with 4% Aptamil BMF was similar at T_0_, T_6_, T_24_, and T_72_, while osmolality of DHM fortified with 4% FM85 and 4% FM85 plus 2% MCT Oil was higher than that of MOM fortified in the same mode at each study point. (Table 3)

**Table 3:** Comparisons of changes in osmolality (mOsm/kg) at T_0_, T_6_, T_24_, and T_72_ of unpasteurized preterm mother’s own milk (MOM) and pasteurized donor human milk (DHM) without fortification or after fortification with 4% PreNAN FM85, 4% PreNAN FM85 and 2% MCT, and 4% Aptamil BMF. Mean ± SD.

	T_0_	T_6_	T_24_	T_72_
Unfortified MOM	297.2 ± 4.8	301.0 ± 1.4	301.6 ± 2.1	300.6 ± 1.1*
Unfortified DHM	298.6 ± 8.4	295 ± 4.1	297.2 ± 5.2	299.2 ± 5.2
P	0.754	0.115	0.117	0.572
MOM plus 4% FM85	396.2 ± 12.7	395.0 ± 11.3	396.2 ± 13.9	401.4 ± 14.2
DHM plus 4% FM85	422.2 ± 17.1	426.6 ± 20.0	431.0 ± 20.5	434.2 ± 22.8
P	0.026	0.015	0.014	0.026
MOM plus 4% FM85 and 2% MCT	389.0 ± 17.3	396.0 ± 18.3	398.0 ± 25.0	402.0 ± 24.0
DHM plus 4% FM85 and 2% MCT	422.8 ± 18.0	430.2 ± 18.6	433.0 ± 22.9	438.4 ± 19.3
P	0.016	0.019	0.049	0.030
MOM plus 4% Aptamil BMF	435.0 ± 6.5	451.2 ± 4.6	447.8 ± 9.2	459.6 ± 8.9
DHM plus 4% Aptamil BMF	426.8 ± 16.0	441.4 ± 15.6	442.6 ± 18.5	446.8 ± 18.2
P	0.319	0.215	0.589	0.120

## Discussion

In our study we found, as expected, that fortified pasteurized DHM and unpasteurized MOM had a higher osmolality than unfortified HM at all study points. Fortification with FM85 increased the osmolality of MOM to a less statistically significant extent than that of DHM, while fortification with Aptamil BMF increased the osmolality of MOM more than FM85 statistically significantly. Moreover, the addition of MCT to FHM did not affect its osmolality.

Our data are reassuring since mean osmolality of DHM and preterm MOM generally remained below the AAP recommended safety cut-off value of 450 mOsm/kg [7]. These results confirm previous studies which investigated the effect of FM85 addition to DHM [12,13] and preterm MOM [14]. Conversely, studies which investigated the effect of fortifiers other than FM85 and Aptamil BMF on osmolality of DHM [6,15] and MOM [15,16] showed an increase of osmolality over 450 mOsm/kg. However, robust evidence from literature does not exist linking feed osmolality to gastrointestinal injury [10] and, despite the old recommendations of AAP [7], it has been reported that the safe osmolarity cut-off level for preterm infants is likely somewhere in the range of 400 to 600 mOsm/L [16].

It is noteworthy that previous studies [12, 14—17], except for Piemontese et al. [13], evaluated changes of osmolality only in the first 24 h after fortification. This evidence of lack of changes allowed fortification of HM once a day rather than before each feed contributing to a decrease in handling of HM and workload of nurses. In our study we did not find a significant increase of osmolality 72 h after fortification both in DHM and in MOM samples, except for MOM fortification with Aptamil BMF which in any case did not cause an exceeding of osmolality safety values [16]. Differently, Piemontese et al. [13] reported an increase of osmolality after fortification both in pasteurized DHM and unpasteurized MOM, but from a clinical point of view these differences were not relevant because also in this study osmolality did not exceed safety values [16].

We observed that fortification with FM85 increased the osmolality of DHM more than that of MOM. This difference could be due to the processing of DHM which includes thawing: it has been reported that thawing increased osmolarity of HM after fortification when compared with unfrozen milk [16]. In fact, it has been reported that a previous freezing step can alter the fat globule structure breaking it down [18]. However, the biochemical mechanism for this effect regarding thawing and osmolality is not fully understood and needs further investigation.

In our study, fortification with Aptamil BMF increased the osmolality of MOM more than FM85. This difference between fortifiers is likely due to their different composition and, in particular, to the higher content of carbohydrates of Aptamil BMF in comparison with FM85. In fact, the amylase in human milk is relatively resistant to storage procedures and can break down the polysaccharides contained in HMF into their constituent mono- and oligosaccharides which induce the rise of HM osmolality [19], and this effect is greater as the carbohydrate content of fortifiers increases.

Supplementation with MCT of DHM and MOM fortified with FM85 was not followed by changes in osmolality. We did not find in literature any studies investigating the possible effects of lipids on HM osmolality. However, MCT fat supplement has been reported to be a very useful calorie-dense source which does not increase the osmolality of HM [20] and our findings confirm this affirmation and are reassuring from this point of view.

## Conclusions

In conclusion, we confirm that fortification with FM85 and Aptamil BMF of pasteurized DHM and unpasteurized preterm MOM increased osmolality in comparison with unfortified HM. We found that this increase remained stable in the 72 h after fortification without exceeding the safety values excluding that changes of osmolality preclude the theoretically possibility of preparing 72 h volumes of FHM. Furthermore, we demonstrated that supplementation of studied FHM with MCT does not change osmolality suggesting increasing energy intake in preterm infants in this way is safe.

## Data Availability

The datasets used and/or analysed during the current study are available from the corresponding author on reasonable request.

## List of abbreviations

DHM: Donor human milk
FHM: Fortified human milk
HM: Human milk
HMF: Human milk fortifiers
MCT: medium-chain triglycerides
MOM: mother’s own milk
